# Safe abortion services for adolescents in Ghana

**DOI:** 10.11604/pamj.2020.35.98.22332

**Published:** 2020-04-06

**Authors:** Luchuo Engelbert Bain

**Affiliations:** 1 Athena Institute for Research on Innovation and Communication in Health and Life Sciences, Vrije Universiteit, Amsterdam, the Netherlands; 2 University of Bordeaux, Inserm, French National Research Institute for Sustainable Development (IRD), Bordeaux Population Health Research Center, Team IDLIC, UMR 1219, Bordeaux, France; 3 Global South Health Research and Services, Liedestraat 9, 1316HE, Almere, the Netherlands

**Keywords:** Safe, abortion, services, Ghana, adolescents

## To the editors of the Pan African Medical Journal

Ghana has one of the most liberal abortion laws in sub-Saharan Africa. The Ghanaian abortion law of 1985 permits abortion in cases of rape, incest or the defilement of a female idiot if the life or health of the woman is in danger; or if there is risk of fetal abnormality. A woman is also legally allowed to obtain an abortion for mental reasons [1]. However, a diagnosis from a psychiatrist is not a mandatory requirement. The challenges in accessing safe abortion services by adolescents are different from those faced by adults. The law, as well as the Ghana Health Service Reproductive Health Strategy, does not provide specific guidance regarding access to safe abortion care for adolescents. Five main areas need special attention as far as improving adolescent access to safe abortion services in Ghana is concerned: (1) Rendering safe abortion fees uniform and affordable in facilities that offer safe abortion care; (2) Establishment of explicit context specific guidelines to fight against stigma among abortion seekers and providers; (3) Training of health care providers in respectful and non-judgmental counseling policies when caring for pregnant adolescents; (4) Expanding the availability and affordability of Long Acting Reversible Contraceptives (LARCs) in health care facilities; (5) Inclusion of clear policies on how to deal with conscientious objection in health facilities.

The more affordable safe abortion fees are, the less likely pregnant adolescents shall turn to unsafe abortion practices [1, 2]. Indeed, the average cost of obtaining a safe abortion in Ghana has been described as unaffordable for an average Ghanaian adolescent [2, 3]. Labelling of girls as bad girls, fear and distrust in health care staff by adolescents, and abortion stigma from the community are some of the factors that reduce access to safe abortion care [2]. Health care providers as well as clients have are victims of abortion related stigma. Antiye et al have suggested an increased overt institutional support for safe abortions as a route to reduce abortion stigma [3]. Long Acting Contraceptives (LARCs), though highly effective and adapted for adolescents and young women, remain grossly unaffordable for adolescents [4]. This should be strongly recommended in the post abortion counseling sessions in order to prevent the frequent recurrent unintended pregnancies reported among persons seeking abortions. Individual, interpersonal, community and policy level reflections are highly needed to set up adolescent friendly, affordable and acceptable safe abortion services in Ghana.
